# Supplementary material for: Microglia control vascular architecture via a TGFβ1 dependent paracrine mechanism linked to tissue mechanics
Source: Nat Commun. 2020 Feb 20;11:986. doi: 10.1038/s41467-020-14787-y (PMC7033106; doi:10.1038/s41467-020-14787-y)
Supplement: Supplementary file 1 — Supplementary File [file 41467_2020_14787_MOESM1_ESM.pdf]

Supplementary Information

Microglia control vascular architecture via a TGFβ1 dependent paracrine mechanism linked to tissue mechanics

Dudiki et al.

Supplementary figure 1

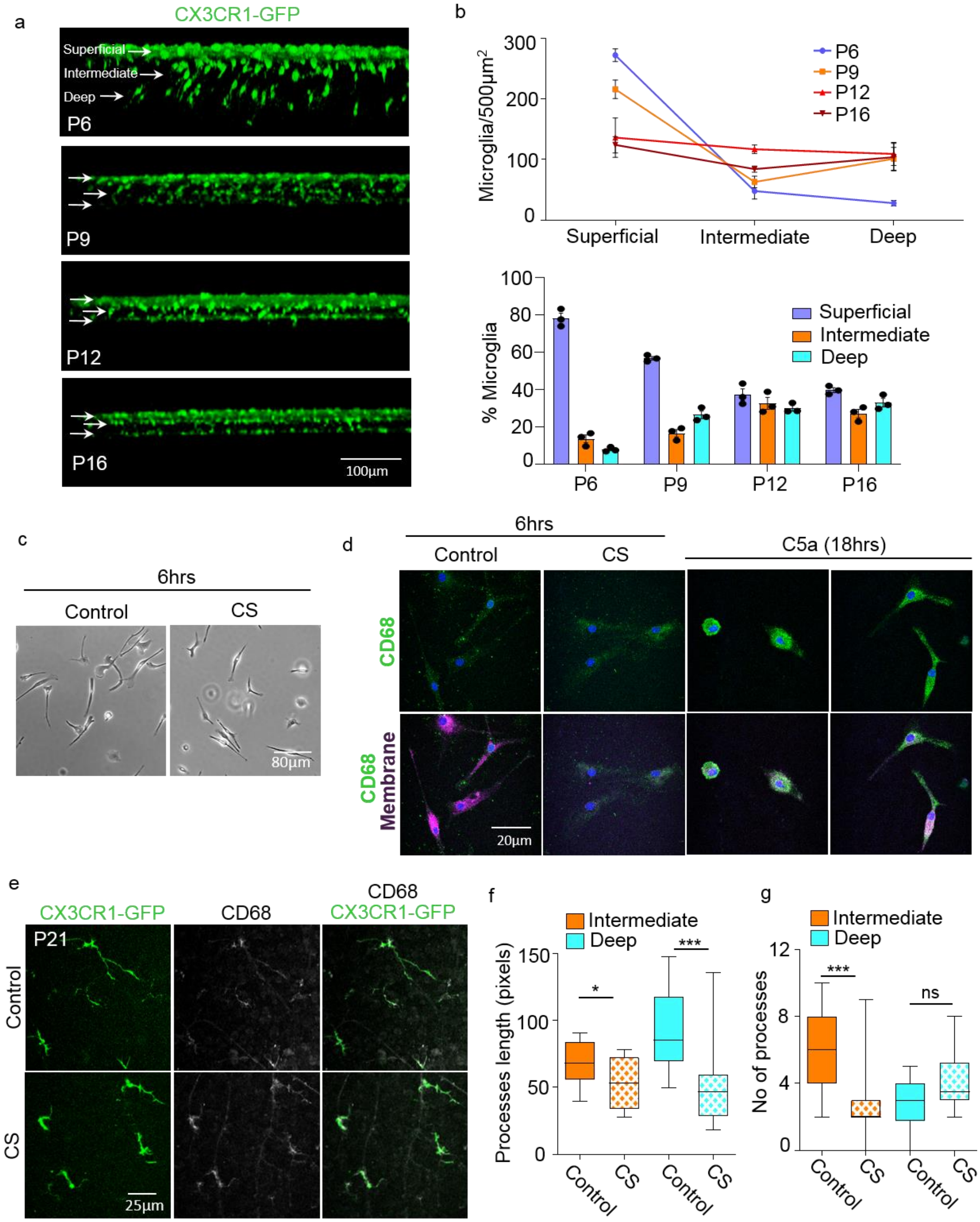

Supplementary Figure 1. Microglial migration in retinal development and response to tissue softening with chondroitin sulfate. a. Confocal images of whole-mount retinas form ages P6 to P16 showing chronological population of deeper retinal layers by CX3CR1-GFP microglia during postnatal retinal development. Note the elongated shape of microglia extending from the superficial layer indicative of migrating microglia at P6. **b.** Quantification of microglial numbers in each retinal layer at different ages shown as a line graph (data represented as mean and error bars are standard deviation (SD)) and bar graphs (data represented as mean and error bars are standard error of mean (SEM)) simultaneously (N=3 mice). At P6, almost all microglia are observed in the superficial layer. By P12, the microglia are distributed evenly among all the layers. **c.** Representative phase contrast images from three experiments depicting the morphology of WT microglia cultured in the presence of 20mg/ml CS or vehicle for 6hrs. Microglia treated with CS appear spread and polarized similar to WT, indicative of resting state. **d.** Representative confocal images of cultured WT microglia stained with CD68 (green), DAPI (blue) and WGA membrane marker (magenta). Microglia were treated with 20mg/ml CS or vehicle alone (control) for 6hrs or with complement protein C5a (known to activate microglia) for 18hrs. Representative images from two independent experiments are shown. **e.** Representative phase contrast images of WT P21 whole mount retinas treated with 20mg/ml CS for up to 6hrs followed by staining with CD68 (gray) (N=3 mice). CS treatment did not increase the expression of microglial activation marker CD68. f,g. Comparison of microglia morphologies in the intermediate and deep layers of 6hrs CS treated and control retinas; major processes length (P=0.0149 (*), P<0.0001 (***); N=16 (intermediate) and 24 (deep) cells from 3 mice) and number of processes (P<0.0001 (***); N=18 (intermediate) and 14 (deep) cells from 3 mice). Statistical significance was calculated using two-tailed *t*-test. Center line of box plots represent the median, bound of box show 25th to 75th percentiles, and upper and lower bounds of whiskers represent the maximum and minimum values, respectively.

Supplementary figure 2

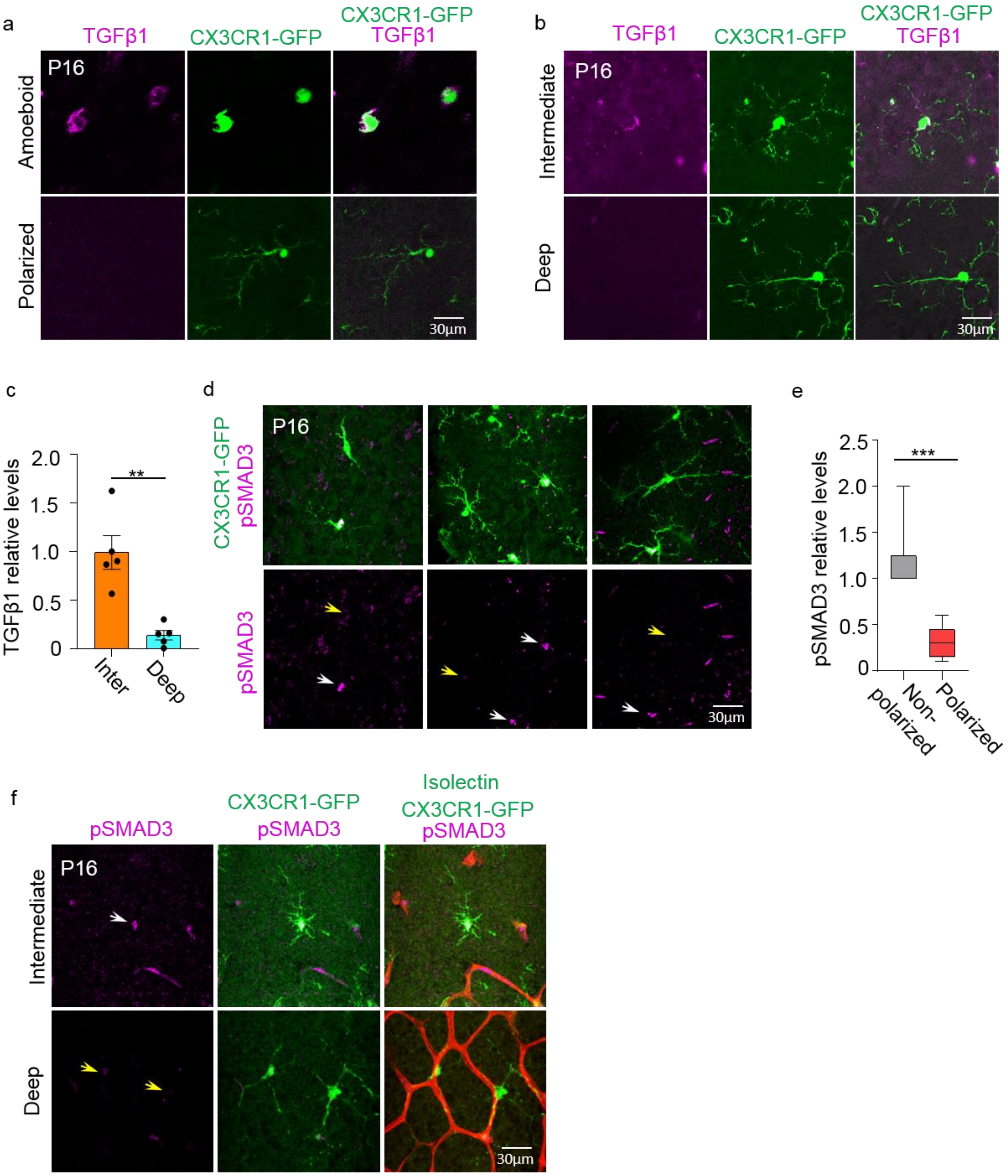

Supplementary Figure 2. TGFβ1 regulation by microglia in P16 retina. a. Representative confocal images from five mice showing high levels of TGFβ1 expression in amoeboid/non-polarized vs polarized microglia in retina. **b**. Ramified/non-polarized microglia of softer intermediate layer expresses substantially more TGFB1 compared to deep microglia polarized on stiff ONL. **c.** Relative TGFB1 levels in microglia of intermediate and deep layer (two-tailed *t*-test; *P*=0.0015 (**); N=5 mice). **d.** Three representative confocal images of pSMAD3 levels in non-polarized (white arrow heads) and polarized (yellow arrow heads) microglia. **e.** Quantification of relative pSMAD3 levels in non-polarized and polarized microglia (two-tailed *t*-test; *P*<0.0001 (***); N=9 cells per group from three mice). **f.** The ramified/non-polarized microglia of the softer intermediate layer show higher pSMAD3 localization to nucleus (white arrow head) compared to deep microglia (yellow arrow heads) polarized on the stiff ONL of retina. A representative image of 4 mice is shown. All bar graphs are represented as mean and error bars are SEM. Center line of box plots represent the median, bound of box show 25th to 75th percentiles, and upper and lower bounds of whiskers represent the maximum and minimum values, respectively.

Supplementary figure 3

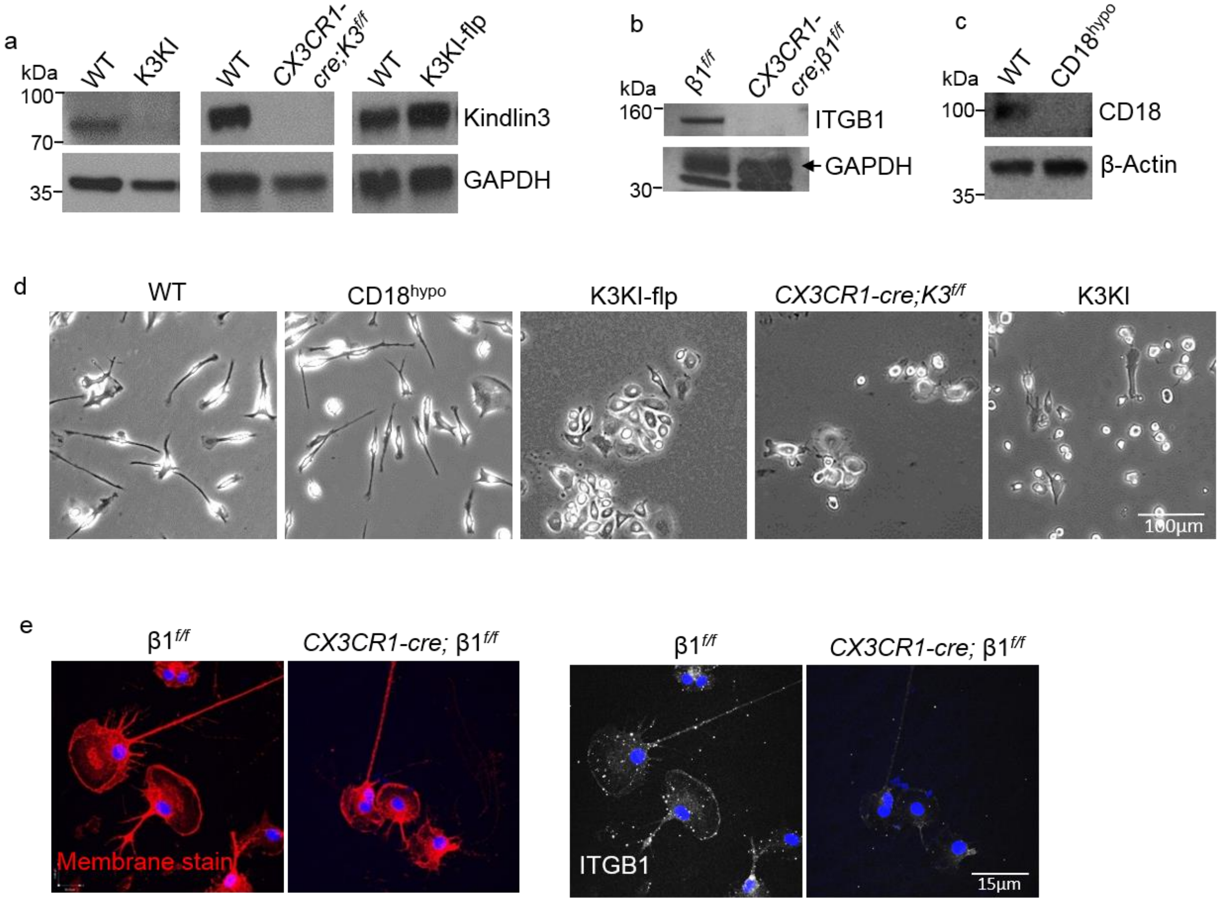

Supplementary Figure 3. Kindlin3 in microglia morphology. a-c. Western blot analysis of primary microglia or bone marrow-derived macrophages (BMDM) for the expression of K3, ITGB1, and CD18, respectively. Primary microglia and BMDM were pooled from four and two mice respectively per genotype and analyzed. Images from two experiments are shown. **d.** Phase-contrast images of primary microglia isolated from integrin and K3-deficient mouse models spread overnight on fibronectin-coated plates. Representative of five experiments. **e.** Primary microglia isolated from β1-integrin knockout mice (*CX3CR1-cre; β1*$^{f/f}$) and control *β1*$^{f/f}$ stained for β1-integrin (ITGB1) after spreading on fibronectin (N=3).

Supplementary figure 4

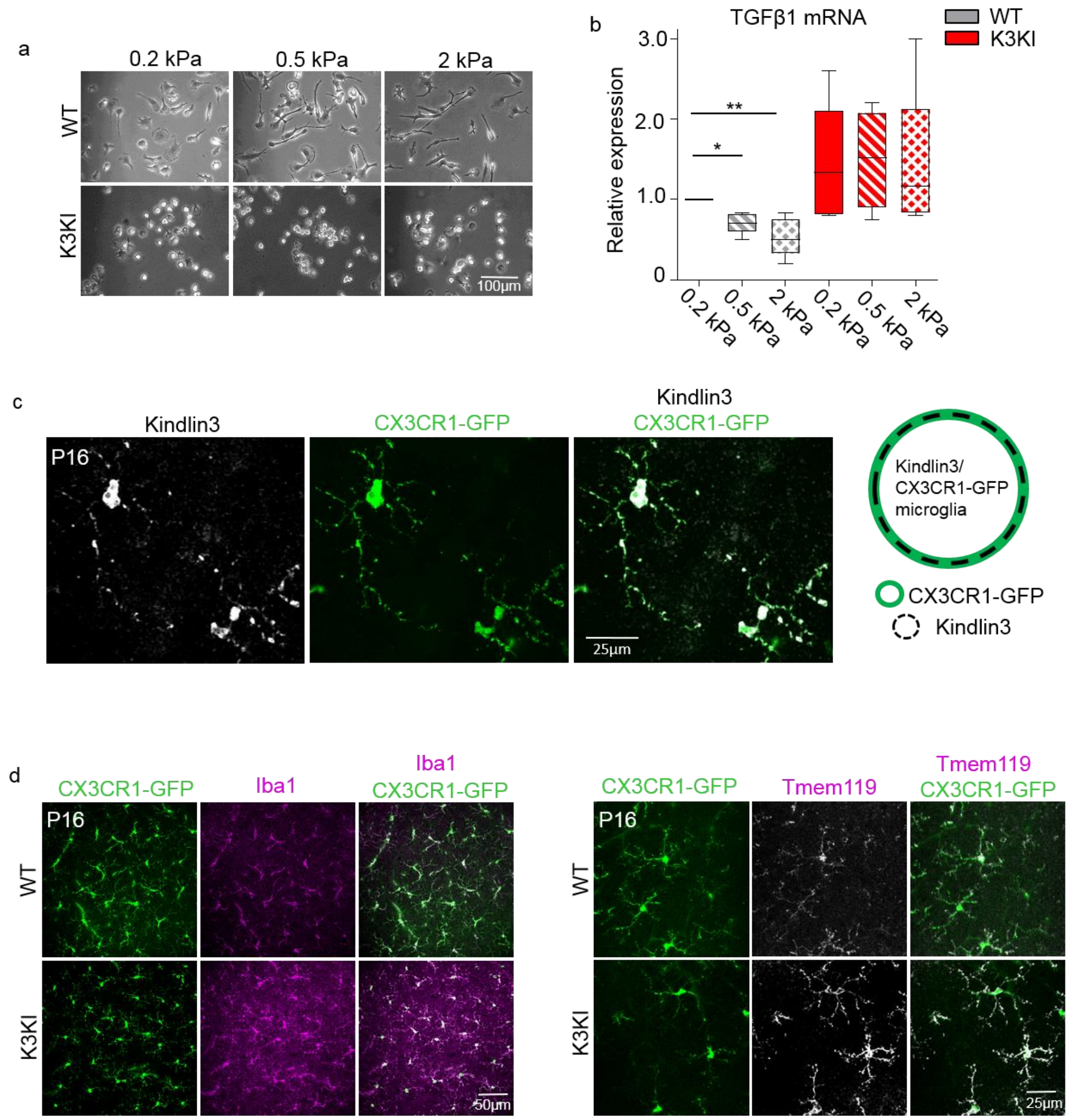

e

	Iba1/ CX3CR1-GFP	Tmem119/ CX3CR1-GFP
WT	97.2±0.03%	95.2±0.02%
K3KI	97.7±0.02%	94.3±0.04%

Supplementary Figure 4. Microglia-specific Kindlin3 is essential for microglial mechanosensing function. a. Representative phase-contrast images of WT and K3KI microglial cells on fibronectin-coated silicone gels of 0.2, 0.5, and 2kPa stiffness (N=5 experiments). **b.** QPCR analysis showing opposite correlation of microglial TGFβ1 mRNA levels with substrate stiffness. No changes were observed in K3KI microglia. Center line of box plots represent the median, bound of box show 25th to 75th percentiles, and upper and lower bounds of whiskers represent the maximum and minimum values, respectively (one-way ANOVA with Dunnett's post-hoc analyses; *P*= 0.0222 (*), *P*=0.0009 (***); N=5 experiments). **c.** Confocal images of whole-mount P16 retinas with CX3CR1-GFP microglia immunostained for K3. K3 exhibits 100% co-localization with CX3CR1-GFP as shown by the Venn diagram. **d.** Confocal images of whole-mount P16 retinas with CX3CR1-GFP microglia co-stained for Iba-1 and Tmem-119. **e.** Quantification of CX3CR1-GFP cells expressing Iba1 and Tmem119 in P16 WT and K3KI retinas. All values are mean and error bars are SEM, N=3 mice. ~98% and 95% of CX3CR1-GFP cells are positive for Iba-1 and Tmem119 respectively in each genotype.

Supplementary figure 5

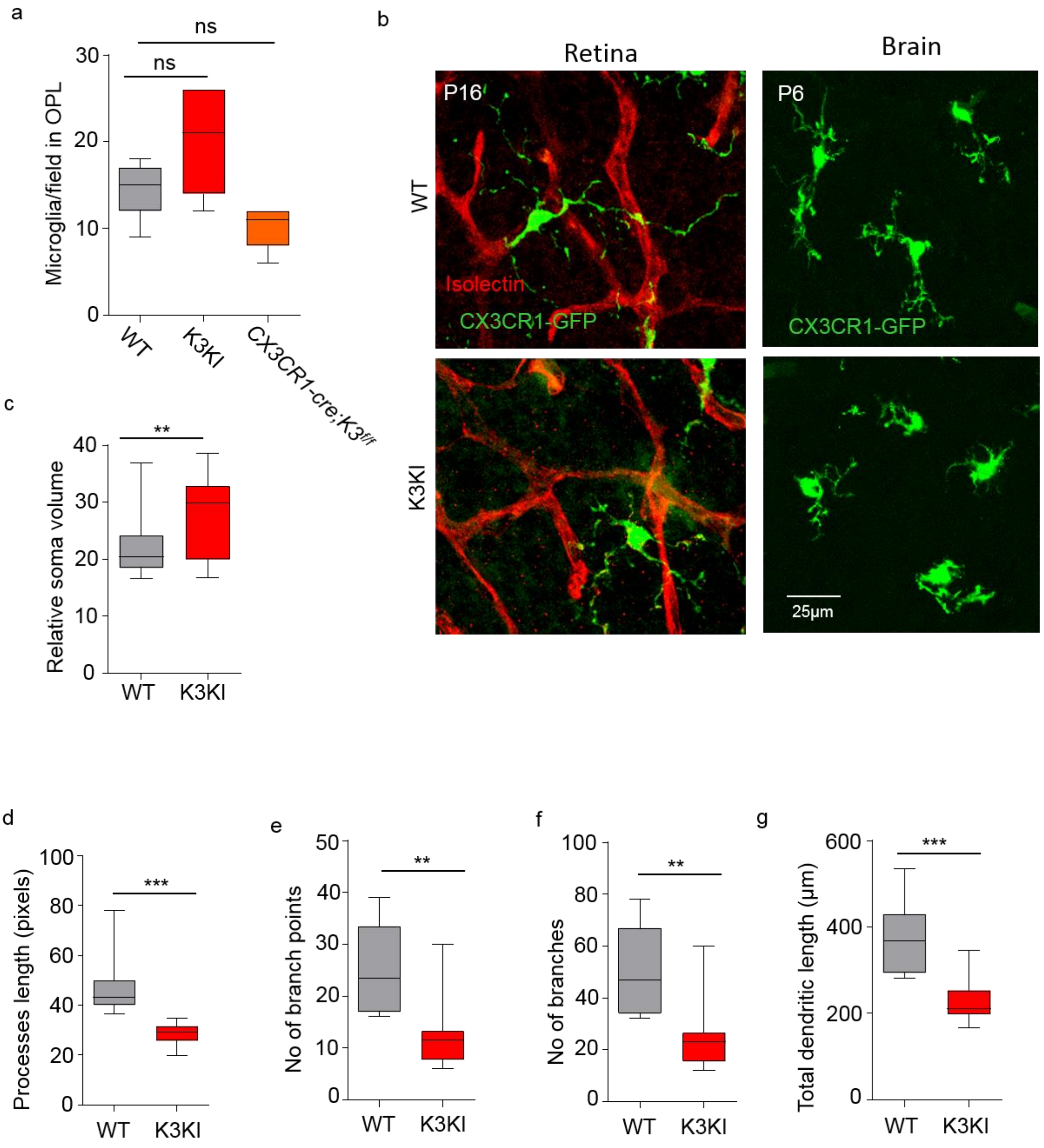

Supplementary Figure 5. Morphological characterization of K3KI microglia. a. Comparison of microglial numbers in the deep layer of WT, K3KI, and K3KO (*CX3CR1-cre;K3*$^{f/f}$) retinas (one-way ANOVA with Dunnett's post-hoc; $P_{WT\text{-}K3KI}$=0.0663 (ns), $P_{WT\text{-}K3KO}$= 0.1320; N=3 mice per group). K3KI at P16 showed a small increase in microglial numbers. However, complete knockout of K3 in *CX3CR1-cre;K3*$^{f/f}$ showed a decrease at P16, likely indicating an indirect consequence of K3 deficiency on microglial numbers. **b.** Confocal images of CX3CR1-GFP microglia in retina and brain. **c-g.** Comparison of the WT and K3KI microglial morphologies; volume of cell soma (*P*=0.0032 (***); N=22 cells from 3 mice) major processes length (*P*<0.0001 (***); N=22 cells from 3 mice), number of branch points (*P*=0.0015 (**); N=10 cells from 3 mice), number of branches (*P*=0.0015 (**); N=10 cells from 3 mice), and total dendritic length (*P*=0.0002 (***); N=10 cells from 3 mice). Statistical significance were calculated using two-tailed *t*-test. Center line of box plots represent the median, bound of box show 25th to 75th percentiles, and upper and lower bounds of whiskers represent the maximum and minimum values, respectively.

Supplementary figure 6

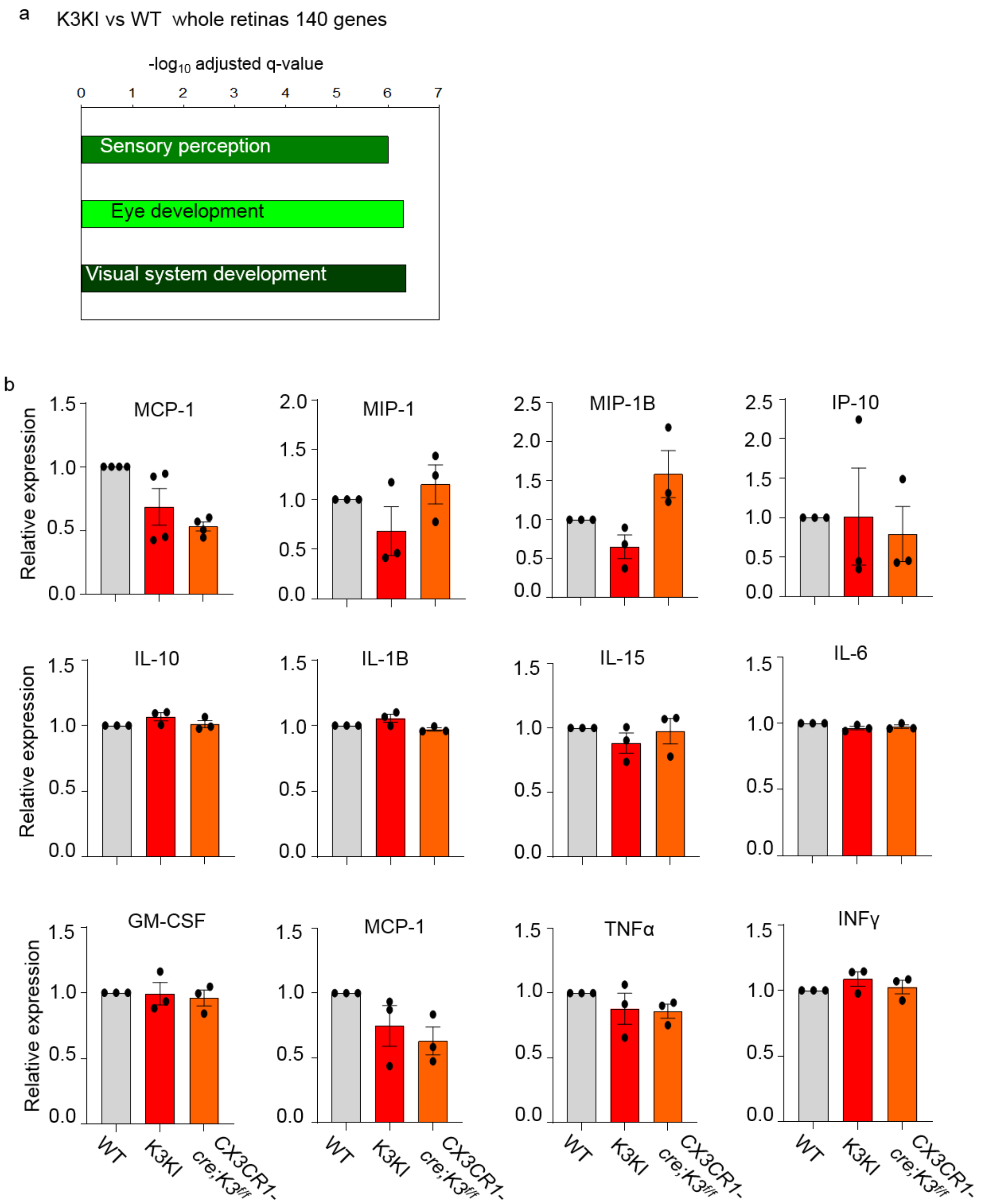

Supplementary Figure 6. Gene and protein expression arrays of microglia. a. Graph summarizing the pathways overrepresented in RNA expression arrays of *CX3CR1-cre;K3*$^{f/f}$ and control 14 day old retinas (N=3 mice per group). The list of genes with significant changes (with p<0.05) was analyzed by ConsensusPathDB at http://cpdb.molgen.mpg.de/ according to the protocol by Herwig *et al*. for over-representation analysis of gene sets. From the resulting analysis top 3 overrepresented pathways (based on q-values calculated by ConsensusPathDB) are shown in the graph. A total of 140 genes, from sensory perception to eye development, were significantly altered. The list of these genes are provided in the source data file. **b.** Results of ELISA-based UPLEX assays for cytokine levels in microglial cell lysates from WT, K3KO, K3KI, and K3KI-flp mice (N= 3 biological replicates), respectively. All bar graphs are represented as mean and error bars are SEM.

Supplementary figure 7

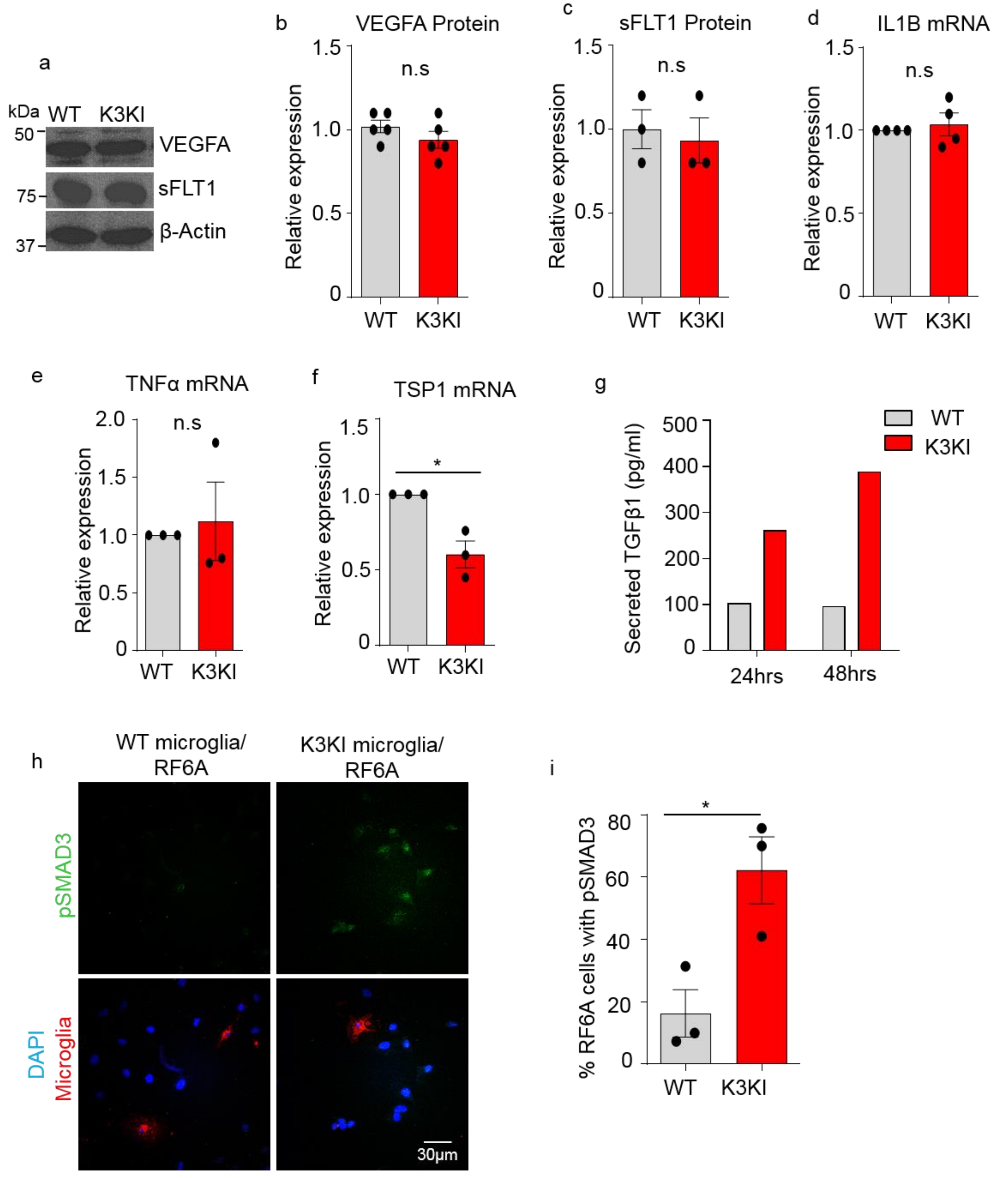

Supplementary Figure 7. Analysis of growth factors and cytokines production by K3KI microglia in comparison to WT. a. A representative western blot of WT and K3KI retinas from five mice for VEGFA and soluble-FLT1 (sFLT1). **b,c.** Densitometry quantification of sFLT and VEGFA levels (two-tailed *t*-test; P=0.2415 (ns); N=5 mice) and sFLT1 (two-tailed *t*-test; P=0.7247 (ns); N=3 mice) from western blot analysis. **d-f.** QPCR analysis of microglia from WT and K3KI mice for IL-1B (P=0.6056 (ns); N=4 experiments), TNFα (P=0.7421 (ns); N=3 experiments), and TSP1 mRNA levels (P=0.0114(*); N=3 experiments). Microglia for each experiment were pooled from four mice. **g**. High levels of latent TGFβ1 secreted by K3KI primary microglia into serum-free media at 24 hr and 48 hr as compared to WT microglia were assessed by ELISA. Microglia were pooled from four mice and a representative bar graph out of two experiments is shown. **h.** Confocal images of RF6A endothelial cells immunostained for pSMAD3. RF6A cells were co-cultured with WT and K3KI microglia in serum-free media for 24 hrs. Cell were than fixed and stained with the microglia-specific marker Iba1 (red), DAPI for all cell nuclei, and pSMAD3 (green). RF6A cells co-cultured with K3KI microglia showed higher pSMAD3 levels, representative of three experiments. **i.** Relative quantification of RF6A cells showing high pSMAD3 levels (P=0.0251 (*); N=3 experiments). Statistical significance was calculated using two-tailed *t*-test. All bar graphs are represented as mean and error bars are SEM.

Supplementary figure 8

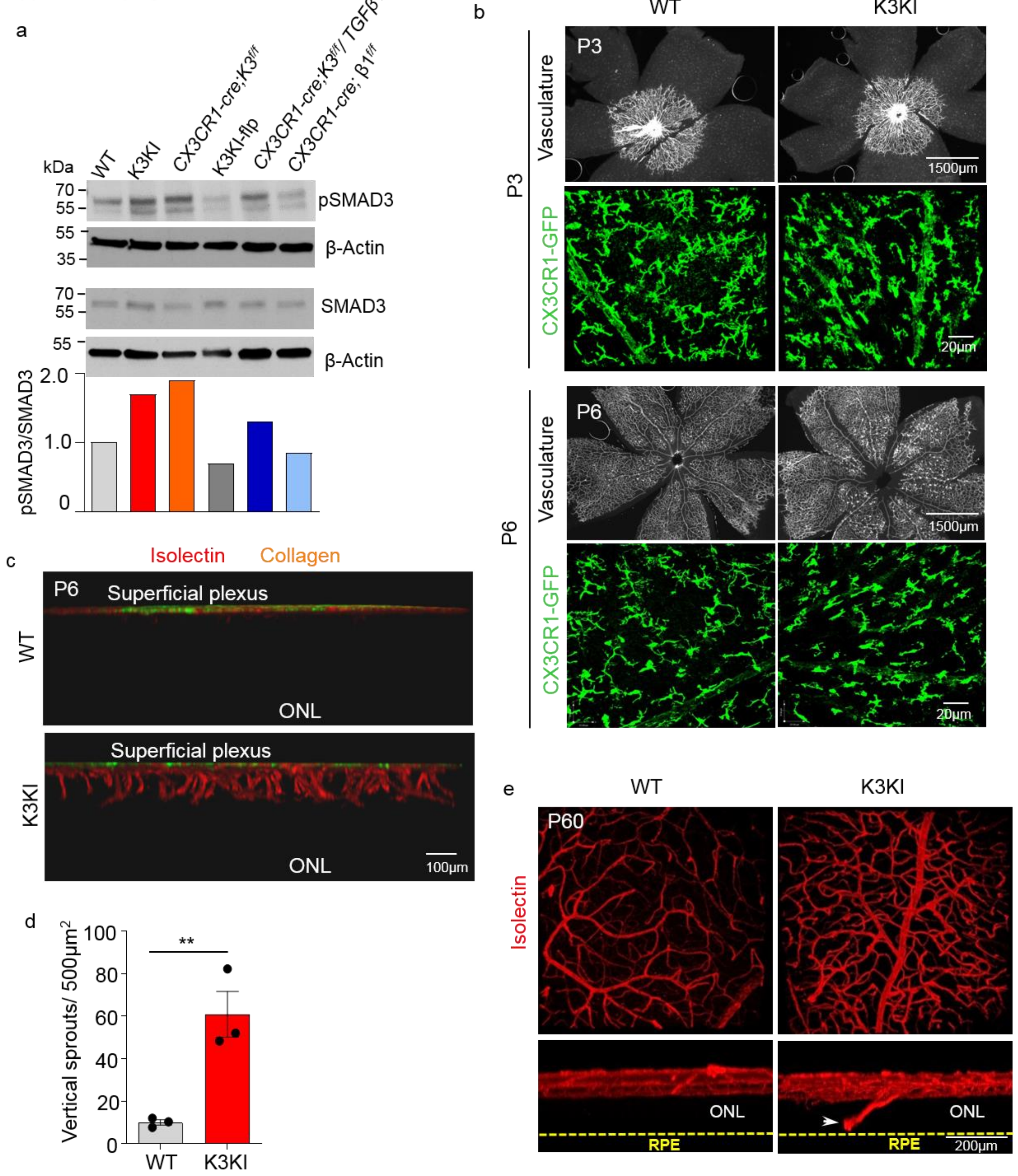

Supplementary Figure 8. High levels of TGFβ1 causes premature vascular sprouting and maturation in K3KI retinas. **a.** Western blots showing high pSMAD3 levels in K3KI and K3KO (*CX3CR1-cre;K3$^{f/f}$*) retinas as compared to WT. Retinas from three mice were pooled and two identical western blots were run and probed with antibodies for pSMAD3 and Smad3, followed by beta-actin for loading control. The pSMAD3/Smad3 levels for the shown western blot were determined after adjusting SMAD3 levels to the actin levels. The K3KI and K3KO retinas show increased pSMAD3 levels compared to WT. At the same time, K3KI-flp knock-in lacking integrin interaction was similar to WT. A significant reduction in pSmad3/Smad3 levels was observed for Kindlin3/TGFβ1 conditional double knockout (*CX3CR1-cre;K3$^{f/f}$/TGFβ1$^{f/f}$*) retinas, indicating efficient deletion of microglial TGFβ1. pSMAD3 levels in β1 microglia-specific KO were similar to those in WT. **b.** Whole-mount retinas at P3 and P6 immunostained with isolectin to show vasculature (white) and microglia expressing CX3CR1-GFP. **c.** 3D reconstitution of whole-mount P6 retina stained with isolectin and collagen showing a cross-sectional view of the vascular layers. Kindlin3-deficient retinas showed premature formation of vertical neo-vascular sprouts from the superficial vascular plexus toward the deeper layers of retina. **d.** Quantitation of the number vertical neo-vascular sprouts per field is shown on the right (two-tailed *t*-test; P=0.0093 (**); N=3 mice). The bar graph represents mean and error bars are SEM. **e.** 3D reconstituted confocal images of P60 retinas stained with isolectin. Only one out of the six K3KI mice analyzed showed sub-retinal vascular lesions (indicated by white arrowheads) originating from the deep vascular plexus and resting on the RPE layer (indicated by the yellow line).

Supplementary figure 9

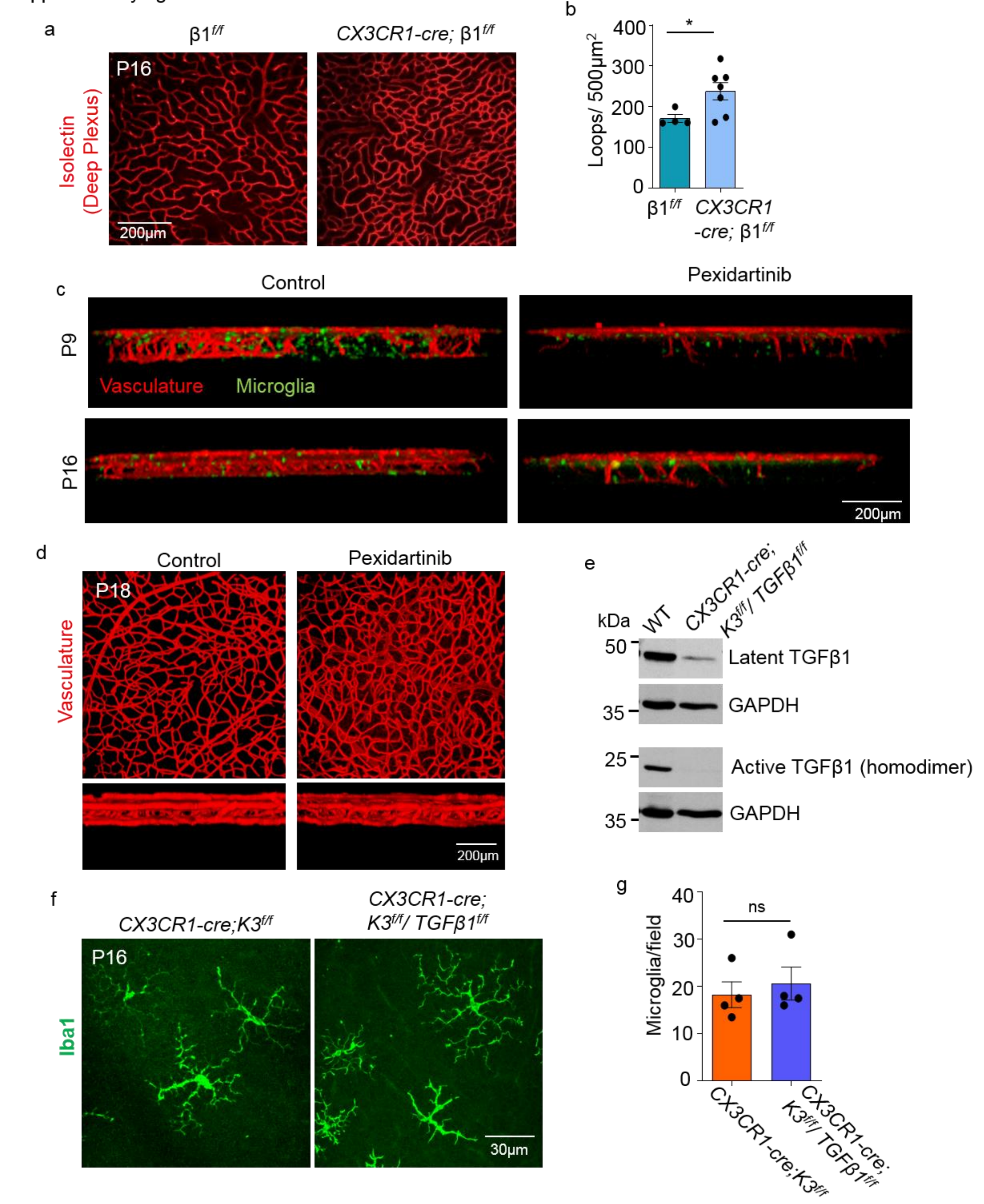

Supplementary Figure 9. Microglia are crucial for retinal vascular development. a. Confocal image of deep vascular plexus from whole-mount retinas of β1 KO (*CX3CR1-cre;* $\beta1^{f/f}$) and control ($\beta1^{f/f}$) mice stained with isolectin. **b**. Bar graphs representing the average loop numbers and loop area of blood vessels in the deep vascular plexus (two-tailed *t*-test; *P*=0.0496 (*); N= 4 $\beta1^{f/f}$ and 7 β1 KO mice). **c.** Microglia depletion with pexidartinib treatment in mice was initiated at P2 and ended at P9 or P16 prior to retinal isolation. 3D reconstitution of whole-mount retinas from P9 and P16 mice treated with pexidartinib or vehicle alone (control). A lateral view is shown to visualize the three vascular plexi (superficial, intermediate, and deep) in red and CX3CR1-GFP-expressing microglia (green). **d.** Pexidartinib treatment in mice starting at P9 and ending at P18 prior to retinal isolation. 3D reconstitution of whole-mount retina from P18 mice treated with pexidartinib or vehicle alone (control). A superimposed and lateral view of the vascular plexi representative of three mice are shown. At P18, the lack of microglia resulted in a modest increase in vascular density. **e.** Western blot showing successful knock out of TGFβ1 from bone marrow-derived macrophages after tamoxifen treatment to *CX3CR1-cre;*$K3^{f/f}/TGF\beta1^{f/f}$ mice. A representative of three mice of each genotype analyzed is shown. **f.** Confocal images representing the morphology of Iba1-immunostained microglia in the retinas of *CX3CR1-cre;*$K3^{f/f}$ and *CX3CR1-cre;*$K3^{f/f}/TGF\beta1^{f/f}$ mice. Representative images from four mice are shown. **g.** Quantification of microglial numbers microglia in the deep layers of retinas from *CX3CR1-cre;*$K3^{f/f}$ and *CX3CR1-cre;*$K3^{f/f}/TGF\beta1^{f/f}$ mice. No significant difference was observed with two-tailed *t*-test; *P*=0.6100 (ns); N=4 mice). All bar graphs are represented as mean and error bars are SEM.

Supplementary figure 10

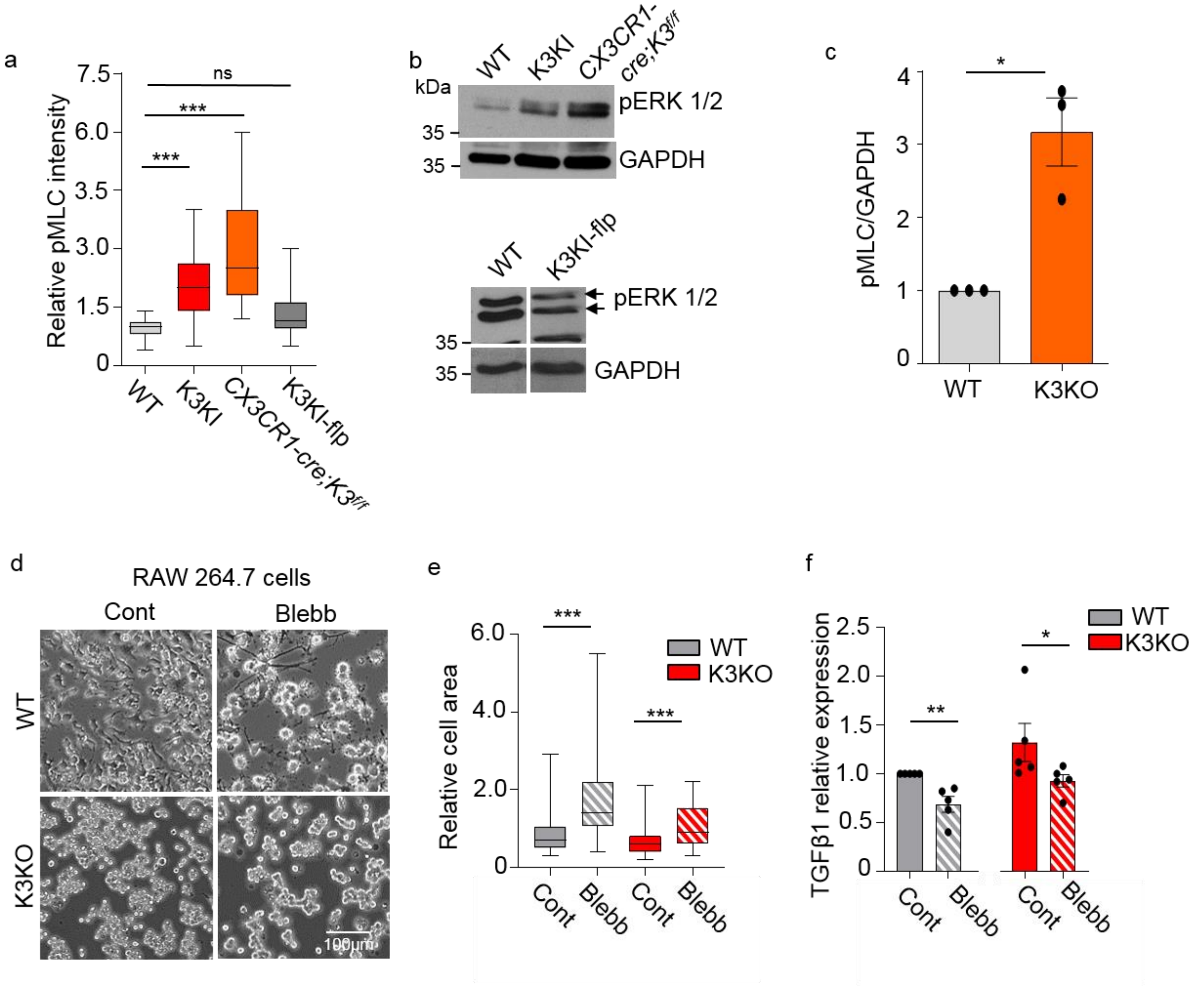

Supplementary Figure 10. Kindlin3 deficiency leads to increased pMLC and pERK. a. Quantification of pMLC levels in WT, K3-deficient (K3KI and *CX3CR1-cre;K3*$^{f/f}$), and K3 mutant (K3KI-flp) immunostained microglia. Significantly higher levels of pMLC were observed only with K3 deficiency (one-way ANOVA with Dunnett's post-hoc; *P*<0.0001 (***), *P*= 0.1922 (ns); N=30 cells per group from three experiments). **b.** Western blot analysis of whole retinas showing increased pERK1/2 levels in K3-deficient, but not K3 mutant (K3KI-flp), mice. GAPDH was used as a loading control. **c.** Quantification of immunoreactive bands from western blot analysis showing high pMLC levels in the two clones of K3KO RAW cells compared to WT after normalization to GAPDH (two-tailed *t*-test; *P*= 0.0214 (*), N=3 experiments). **d.** Phase-contrast image of K3KO RAW cells treated with DMSO (control) or 50μM blebbistatin (Blebb) and spread on fibronectin-coated plates for 12 hrs. Representative of three experiments. **e.** Bar graph shows cell spread area relative to WT control (two-tailed *t*-test; *P*<0.0001 (***); N=78 cells per group from three experiments) **f.** QPCR analysis for TGFβ1 in control vs. blebbistatin-treated RAW cells (one-tailed *t*-test; *P*=0.00090 (**), *P*=0.0493 (**); N=5 experiments). Center line of box plots represent the median, bound of box show 25th to 75th percentiles, and upper and lower bounds of whiskers represent the maximum and minimum values, respectively. All bar graphs are represented as mean and error bars are SEM.

Supplementary Methods

Table 1. Key resources used in the study

Reagent	Company	Catalog #	Notes
C57BL/6	Jackson Laboratory	000664	Mouse
Cx3cr1$^{GFP/GFP}$	Jackson Laboratory	5582	Mouse
Cx3cr1-cre (inducible)	Jackson laboratory	21160	Mouse
TGF β1 $^{flox/flox}$	Jackson Laboratory	010721	Mouse
Integrin β1 $^{flox/flox}$	Jackson Laboratory	4605	Mouse
CD18 hypomorph	Jackson Laboratory	2128	Mouse
Tamoxifen Powder	Sigma-Aldrich	T5648	
Chondroitin Sulfate	Selleck Chemicals	S2416	
PFA	Electron Microscope Sciences	15713	
Triton 100X	Bio-Rad	161-0407	
Sucrose	Sigma-Aldrich	S9378	
4-hydroxytamoxifen (4HT)	Sigma-Aldrich	SML1666	
Corn oil	Sigma-Aldrich	C8267	
5% nonfat dry milk	Cell Signaling	9999s	
DMEM/F12		CCF Media core	DMEM:F12 (15mM HEPES, L-glut
Microglia media			DMEM/F12 with 20% FBS, 100 u/ml penicillin and streptomycin, 0.25ug/ml amphotericin B and supplementation of non-essential amino acids (NEAA)
Penicillin/streptomycin (100 u/ml)		CCF Media core	
Amphotericin B		CCF Media core	
Non-essential amino acids (NEAA)	Thermo Fisher Scientific	242572	
Blocking solution			5% goat serum, 3% BSA in 1x PBS

RIPA Buffer	Thermo Fisher Scientific	89900	RIPA lysis and extraction buffer
Fetal Bovine Serum (FBS)	Atlanta Biologicals	S11150H	
Goat serum	Fisher Scientific	ICN2939249	
Bovine Serum Albumin (BSA)	Fisher Scientific	BP-1600-100	
4x Laemmli Buffer	Bio-Rad	1610747	
Nanodrop spectrometer	Nanodrop technologies	ND-1000	
Mini-Protean II system	Bio-Rad		
Confocal microscope	Leica	SP5 confocal/multi-photon microscope	
Cryotome	Leica	CM1850	
Inverted optical microscope	Nikon	Eclipse Ti- TR 400	
Anti-TGFB1	Torrey Pines Biolabs	TP-254	Purified Rabbit Anti-porcine TGFβ1
TMEM119	Abcam	ab209064	Rabbit monoclonal Anti-TMEM119 antibody [28-3] - Microglial marker
α Tubulin	Santa Cruz	sc 8035	Mouse Monoclonal IgM (kappa light chain)
pSMAD3	Abcam	Ab52903	Rabbit monoclonal Anti-Smad3 (phospho S423 + S425) antibody [EP823Y]
SMAD3	Cell Signalling Technologies	9513s	SMAD3 antibody
Iba-1	Wako Chemicals USA	019-19741	Anti Iba1, Rabbit Polyclonal (for Immunocytochemistry)
CD68	Abcam	Ab31630	Anti-CD68 antibody [ED1]
pMLC	Cell Signaling Technologies	3671S	Rabbit Polyclonal anti-p-myosin Light chain
pERK1	Cell Signaling Technologies	9101S	Rabbit Polyclonal anti-Pospho-p44/42 MAPK (Erk1/2) Thr202/Tyr204) Antibody
Actin	Cell Signaling	4967S	Rabbit Polyclonal anti-□□Actin antibody
Phospho-Myosin Light Chain 2 (Ser19)	Cell Signaling	3671	Rabbit Polyclonal anti-Phospho-Myosin Light Chain 2 (Ser19) Antibody

Alexa Fluor® 568 Phalloidin	Thermo Fisher Scientific	A12380	High-affinity F-actin probe conjugated to our superior Alexa Fluor® 568 dye
Wheat Germ Agglutinin (WGA) Alexa Fluor 647 conjugate	Thermo Fisher Scientific	W32466	Alexa Fluor® 647 WGA for labeling of *N*-acetylglucosaminyl and sialic acid residues of glycoproteins on cell surfaces
Isolectin GS-IB4, 568	I21412	ThermoFisher Scientific	The red fluorescent Alexa Fluor® 568 isolectin GS-IB_4 conjugate used for specifically labeling endothelial cells
Goat anti-Rabbit IgG (H+L) Cross-Adsorbed Secondary Antibody, Alexa Fluor 488	A-11008	ThermoFisher Scientific	Goat Polyclonal Secondary Antibody
Goat anti-Rabbit IgG (H+L) Highly Cross-Adsorbed Secondary Antibody, Alexa Fluor 647	A-21245	ThermoFisher Scientific	Goat Polyclonal Secondary Antibody
Goat anti-Rabbit IgG (H+L) Cross-Adsorbed Secondary Antibody, Alexa Fluor 568	A-11001	ThermoFisher Scientific	Goat Polyclonal Secondary Antibody
Goat anti-Mouse IgG (H+L) Cross-Adsorbed Secondary Antibody, Alexa Fluor 568	A-11004	ThermoFisher Scientific	Goat Polyclonal Secondary Antibody
Goat anti-Mouse IgG (H+L) Cross-Adsorbed Secondary Antibody, Alexa Fluor 488	A-11011	Thermofisher Scientific	Goat Polyclonal Secondary Antibody
Anti-rabbit IgG, HRP-linked Antibody	7074S	Cell Signaling Technology	HRP-linked secondary antibody
Anti-mouse IgG, HRP-linked Antibody	7076S	Cell Signaling Technology	HRP-linked secondary antibody
TGFB1 Forward Primer	Integrated DNA Technologies	5’GCGGACT ACTATGCTA AAGAGG3’	Real-Time PCR (qPCR)
TGFB1 Reverse Primer	Integrated DNA Technologies	5’GTTGCTCC ACACTTGAT TTT3’	Real-Time PCR (qPCR)

GAPDH Froward Primer	Integrated DNA Technologies	5’ACTCCCAC TCTTCCACC TTC3’	Real-Time PCR (qPCR)
GAPDH Reverse Primer	Integrated DNA Technologies	5’TCCAGGG TTTCTTACT CCTTG3’	Real-Time PCR (qPCR)
IL-1B Forward Primer	Integrated DNA Technologies	5’ACGGACC CCAAAAGA TGAAG3’	Real-Time PCR (qPCR)
Il-1B Reverse Primer	Integrated DNA Technologies	5’CACGGGA AAGACACA GGTAG3’	Real-Time PCR (qPCR)
TNFα Forward Primer	Integrated DNA Technologies	5’TGGAGTC ATTGCTCTG TGAAG3’	Real-Time PCR (qPCR)
TNFα Reverse Primer	Integrated DNA Technologies	5’CCTGAGC CATAATCCC CTTTC3’	Real-Time PCR (qPCR)
TSP1 Forward Primer	Integrated DNA Technologies	5’GCAGACA CAGACAAA AACGGGGA G3’	Real-Time PCR (qPCR)
TSP1 Reverse Primer	Integrated DNA Technologies	5’TCTCCAAC CCCATCCAT GTCC3’	Real-Time PCR (qPCR)
sgRNA ; K3KO Forward	Integrated DNA Technologies	CACCGACG GGGGAGTC GCACATTGG	
sgRNA ; K3KO Reverse	Integrated DNA Technologies	AAACCCAAT GTGCG ACTCCCCCG TC	
sgRNA ; K3KO2 Forward	Integrated DNA Technologies	CACCGACA GACGTGTGC TGCGGCTT	
sgRNA ; K3KO2 Reverse	Integrated DNA Technologies	AAAC AAGCCGCA GC AACGTCTGT C	
Volocity software	PerkinElmer		
FIJI Image J software	National Institute of Health	Version 1.51K	
ImagePro plus software	Media Cybermetics		
Quant studio 3	Applied Biosystems		
ABI SDS v2.1	Applied Biosystems		

12% polyacrylamide slab gel	Bio-Rad	4561046	12% Mini-PROTEAN® TGX™ Precast Protein Gels, 15-well, 15 µl
Immunobilon-P PVDF membrane	Millipore	123988	Membrane, PVDF, 0.45 µm, 26.5 cm x 3.75 m roll
OCT	Fisher Scientific	23-730-571	Tissue-Plus O.C.T. Compound
Protease inhibitor cocktail	Roche	5892970001	complete™ ULTRA Tablets, Mini, EASYpack Protease Inhibitor Cocktail
mirQury RNA isolation kit	Exiqon (discontinued)	181241	
RT-PCR kit	Qiagen	205111	Omniscript RT kit
Random Hexamers	Fisher Scientific	N8080127	
iQ SYBR Green super mix	BioRad	1708882	
Signalfire ECL kit	Cell signaling Technologies	6883S	
Prolong Gold Antifade Mountant	Thermo Fisher Sientific	P36930	
Hoechst	Invetrogen	H3570	Nuclear staining
Vectashield Mounting medium w/DAPI	Vector	2E0327	w/DAPI (for Nuclear staining)
TGFB1 Emax immune assay system	Promega	G7590	
LentiCRISPRv2 vector	Addgene		
BsmBI	Fermentas		
Pheonix Packaging Cells	Takara		
Lipofectamine3000	Thermo Fisher Scientific	L3000008	
Lenti-X Packaging Single Shots	Clontech		
Puromycin	Santa Cruz	Sc-108071	Puromycin dihydrochloride
Plvx-Dsred-monomer-c1 vector	Clontech	632153	
T4 ligase	NEB	M0202S	
Raw 264.7	ATCC		Macrophage-like, Abelson leukemia virus transformed cell line derived from BALB/c mice.
