# Supplementary material for: Microglia control vascular architecture via a TGFβ1 dependent paracrine mechanism linked to tissue mechanics
Source: Nat Commun. 2020 Feb 20;11:986. doi: 10.1038/s41467-020-14787-y (PMC7033106; doi:10.1038/s41467-020-14787-y)
Supplement: Supplementary file 3 — Reporting Summary [file 41467_2020_14787_MOESM3_ESM.pdf]

nature research

Corresponding author(s): Tatiana V Byzova

Last updated by author(s): Jan 21, 2020

Reporting Summary

Nature Research wishes to improve the reproducibility of the work that we publish. This form provides structure for consistency and transparency in reporting. For further information on Nature Research policies, see Authors & Referees and the Editorial Policy Checklist.

Statistics

For all statistical analyses, confirm that the following items are present in the figure legend, table legend, main text, or Methods section.

n/a	Confirmed	
☐	☒	The exact sample size (*n*) for each experimental group/condition, given as a discrete number and unit of measurement
☐	☒	A statement on whether measurements were taken from distinct samples or whether the same sample was measured repeatedly
☐	☒	The statistical test(s) used AND whether they are one- or two-sided<br>*Only common tests should be described solely by name; describe more complex techniques in the Methods section.*
☐	☒	A description of all covariates tested
☐	☒	A description of any assumptions or corrections, such as tests of normality and adjustment for multiple comparisons
☐	☒	A full description of the statistical parameters including central tendency (e.g. means) or other basic estimates (e.g. regression coefficient) AND variation (e.g. standard deviation) or associated estimates of uncertainty (e.g. confidence intervals)
☐	☒	For null hypothesis testing, the test statistic (e.g. *F*, *t*, *r*) with confidence intervals, effect sizes, degrees of freedom and *P* value noted<br>*Give P values as exact values whenever suitable.*
☒	☐	For Bayesian analysis, information on the choice of priors and Markov chain Monte Carlo settings
☒	☐	For hierarchical and complex designs, identification of the appropriate level for tests and full reporting of outcomes
☒	☐	Estimates of effect sizes (e.g. Cohen's *d*, Pearson's *r*), indicating how they were calculated

Our web collection on statistics for biologists contains articles on many of the points above.

Software and code

Policy information about availability of computer code

Data collection	ABI SDS v2.1 software, Volocity 6.5, LAS X software, QCapture 2.9.12, Igor Pro 6.37 software
Data analysis	Volocity 6.5, FIJI Image J JAVA 6, Image-pro plus 6/7, Graph pad Prism 8, Affymetrix's Transcriptome Analysis Software, ConsensusPathDB, Igor Pro 6.37 software, Excel,

For manuscripts utilizing custom algorithms or software that are central to the research but not yet described in published literature, software must be made available to editors/reviewers. We strongly encourage code deposition in a community repository (e.g. GitHub). See the Nature Research guidelines for submitting code & software for further information.

Data

Policy information about availability of data

All manuscripts must include a data availability statement. This statement should provide the following information, where applicable:

- Accession codes, unique identifiers, or web links for publicly available datasets
- A list of figures that have associated raw data
- A description of any restrictions on data availability

All data generated or analysed during this study are included in this published article (and its supplementary information files). Raw RNA array data will be deposited into the NIH open access repository GEO upon acceptance of the manuscript.The source data for Figures 1d, 1f-i, 2b-d, 2f, 2h-i, 2k-m, 2o-p, 3d-e, 3g, 4b, 4d, 4f-g, 4i, 4k, 5b-e, 5i, 6b-c, 6e-f, 6h, 7a-c, 7e-f, 7h-i and supplementary Figures 1b, 1f, 1g, 2c, 2e, 3a-c, 4b, 4e, 5a, 5c-g, 6a, 6b, 7a-g, 7i, 8a, 8d, 8b, 9e, 9g, 10a-c, 10e-f are provided as a Source Data File.

Field-specific reporting

Please select the one below that is the best fit for your research. If you are not sure, read the appropriate sections before making your selection.

☒ Life sciences ☐ Behavioural & social sciences ☐ Ecological, evolutionary & environmental sciences

For a reference copy of the document with all sections, see nature.com/documents/nr-reporting-summary-flat.pdf

Life sciences study design

All studies must disclose on these points even when the disclosure is negative.

Sample size	1. For all animal studies we estimated the sample size using the formula $n=1+2C(s/d)^2$, where C=7.85 (±/significance 5%, ^{2}/power 80%), with our past standard deviation being 0.717 and reported effect size being 0.659.10 animals/group that would detect a 20-30% difference in means with a power of 0.8 and alpha = 0.05.<br>2. The main phenotype, i.e. neovascularization defect in K3KI mice was confirmed in >22 mice that is well beyond the statistically estimated sample size in our efforts to define the kinetics of vascularization and perform the analysis of various parameters. The phenotype is present in all analyzed mice, it is a rather "yes" or "No" phenomenon and no animals were excluded.<br>3. To confirm the main phenotype and its features, in addition to K3KI mice (Kindlin3 knockin (QW mutations) mice the very same phenotype was confirmed in an independently generated mouse line, namely Kindlin 3 inducible knockout in microglia (CX3CR1 promoter), (n=7). All analyzed mice were positive for excessive sprouts, no exclusions.<br>4. The rescue of neovascular defects was confirmed in microglia-specific (CX3CR1-dreiven) Kindlin3/TGFB1 knockout mice, n=7. All analyzed mice were negative for excessive sprouts, no exclusions.<br>5. For in vitro studies, primary microglia of a specific genotype were isolated from 4 mice of the same age, pooled and used for experiments. The experiments were repeated a minimum of 3 times.
Data exclusions	No data were excluded from analyses
Replication	1. All experiments were performed more than 3 times (as specified in the legends) using animals from multiple breeders.<br>2. The main characteristics of the phenotype were described by at least 2 fellows working at the lab consequently and independently with identical results. The same is true for in vitro experiments which were replicated by at least 2 members of the lab.<br>3. Whenever possible, results obtained using primary microglia from K3KI and K3 KO (conventional knock in and knockouts) were validated using 2 independent CRISPR Kindlin 3 knockout lines.<br>3. To replicate our results we also used multiple mouse lines and rescue experiments. We used K3 knockin mice with point mutations to avoid possible compensatory effects often observed in knockouts. The results were replicated using inducible and tissue-specific K3 KO with all appropriate controls for the side effects of tamoxifen. Thus, the abnormal vascular phenotype of K3KI knockin mice mice was reproduced in a second mouse model with microglia specific inducible knockout of Kindlin3 (CX3CR1-cre;K3fl/fl).<br>4. First characterization of K3 excision and validation of its effect on microglia was performed as described in Meller et all, JCI Insight. For this, primary microglia was isolated from CX3CR1-cre;K3f/f mice and infected with adenovirus encoding Cre-recombinase to initiate Kindlin3 excision as described by us previously in Meller. et al. In our experiments, the functional effects of K3 deletion always phenocopied defects described in Kindlin 3-deficient patients (Malinin et all, Nat.Med, 2009) and Kindlin 3 deficient cell lines. To confirm that the phenotype is a result of Kindlin 3 deficiency but not any "passenger" mutations, we routinely perform a "rescue" with a full length Kindlin3 (as described in Malinin et al, 2009) in our in vitro experiments.<br>5. We rescue the abnormal vascular phenotype using K3 and TGFB1 double knockout mice. In this model, the efficiency of TGFB1 knockout was validated based on the significant decrease in pSMAD3 staining in vivo.
Randomization	In the study we performed morphological analysis and phenotyping of retinas, quantified and analyzed cell morphology, western blots, tissues staining, q-PCR, RNAseq, ELISA. Cellular experiments were performed using cultured cells. Evaluation of phenotype, tissue staining and cellular and vascular morphology was based on at least three randomly selected fields.<br>No pre-selection was used.
Blinding	The data quantitation/analysis was performed by personnel blind to the main hypothesis and the nature/genotype of the samples.

Reporting for specific materials, systems and methods

We require information from authors about some types of materials, experimental systems and methods used in many studies. Here, indicate whether each material, system or method listed is relevant to your study. If you are not sure if a list item applies to your research, read the appropriate section before selecting a response.

Materials & experimental systems

n/a	Involved in the study	
☐	☒	Antibodies
☐	☒	Eukaryotic cell lines
☒	☐	Palaeontology
☐	☒	Animals and other organisms
☒	☐	Human research participants
☒	☐	Clinical data

Methods

n/a	Involved in the study	
☒	☐	ChIP-seq
☒	☐	Flow cytometry
☒	☐	MRI-based neuroimaging

Antibodies

Antibodies used

pSMAD3: Abcam, Ab52903, Rabbit monoclonal Anti-Smad3 (phospho S423 + S425) antibody [EP823Y]
SMAD3: Cell Signaling Technologies, 9513s, Rabbit Polyclonal
pMLC: Cell Signaling Technologies, 3671S Rabbit Polyclonal anti-p-myosin Light chain
pERK1/2: Cell Signaling Technologies, 9101S, Rabbit Polyclonal anti-Pospho-p44/42 MAPK (Erk1/2) Thr202/Tyr204) Antibody
Anti-TGFB1: Torrey Pines Biolabs, TP-254, Purified Rabbit Anti-porcine TGFβ1
TMEM119: Abcam, ab209064, Rabbit monoclonal Anti-TMEM119 antibody [28-3]
Iba-1: Wako Chemicals, USA 019-19741, Anti Iba1 Rabbit Polyclonal
Kindlin3: Abcam, ab68040, Rabbit polyclonal to URP2/Kindlin-3
CD18: Abcam, ab119830, Rat monoclonal [M18/2] to CD18
GAPDH: Abcam, ab181603, Rabbit monoclonal [EPR16884] to GAPDH - Loading Control
B-Actin: Cell Signaling Technologies, 4967s, Rabbit Polyclonal to amino-terminal residues of human β-actin
α Tubulin: Santa Cruz, sc 8035, Mouse Monoclonal IgM (kappa light chain)
CD68 Abcam, ab31630, Anti-CD68 antibody (ED1)
Goat anti-Rabbit IgG (H+L) Cross-Adsorbed Secondary Antibody, Alexa Fluor 488: ThermoFisher Scientific,A-11008
Goat anti-Rabbit IgG (H+L) Highly Cross-Adsorbed Secondary Antibody, Alexa Fluor 647, ThermoFisher Scientific, A-21245
Goat anti-Rabbit IgG (H+L) Cross-Adsorbed Secondary Antibody, Alexa Fluor 568: ThermoFisher Scientific,A-11004, A-11011
Anti-rabbit IgG, HRP-linked Antibody, Cell Signaling Technology, 7074S
Anti-mouse IgG, HRP-linked Antibody, Cell Signaling Technology, 7076S

Validation

pSMAD3: Abcam provides several references for validation. Nevertheless, we validated the antibody by simultaneous analysis of western blots (to confirm protein size and specificity) and immunohistochemistry (to confirm localization to nucleus). Further, significant decrease in pSMAD3 levels was observed inTGFB1 knockout mice, providing an independent confirmation of pSMAD3 specificity. Reference: Flora P et al. Transient transcriptional silencing alters the cell cycle to promote germline stem cell differentiation in Drosophila. Dev Biol 434:84-95 (2018)

SMAD3:Cell signaling provides several references for validation. We validated by western blot analysis (size and specificity). Ref: Hilt, Z. T. et al. Platelet-derived beta2M regulates monocyte inflammatory responses. JCI Insight 4, doi:10.1172/jci.insight.122943 (2019).

pMLC: Cell signaling provides several references for validation. We validated by simultaneously analyzing western blots (size and specificity) and immunohistochemistry (specific localization within the cell). Ref: Lee, W. et al. Dispersible hydrogel force sensors reveal patterns of solid mechanical stress in multicellular spheroid cultures. Nat Commun 10, 144, doi:10.1038/s41467-018-07967-4 (2019)

pERK1/2: Cell signaling provides several references for validation. We validated by simultaneously checking with ERK inhibitor U0126 known to inhibit ERK activity in RAW 264.7 cell line followed by western blot analysis and immunohistochemistry (localization in cell). Reference: Kreis, P. et al. ATM phosphorylation of the actin-binding protein drebrin controls oxidation stress-resistance in mammalian neurons and C. elegans. Nat Commun 10, 486, doi:10.1038/s41467-019-08420-w (2019).

Anti-TGFB1: We validated by checking with western blot analysis (size). Reference: Grande, J. P. Role of transforming growth factor-beta in tissue injury and repair. Proc Soc Exp Biol Med 214, 27-40 (1997).

TMEM119: Abcam provides several references for validation. We validated by immunohistochemistry i.e co-localization of staining with CX3CR1-GFP expressing microglia in retina. Reference: Bennett, M. L. et al. New tools for studying microglia in the mouse and human CNS. Proc Natl Acad Sci U S A 113, E1738-1746, doi:10.1073/pnas.1525528113 (2016).

Iba-1: Wako chemicals provides several references for validation. We validated by immunohistochemistry i.e co-localization of staining with CX3CR1-GFP expressing microglia in retina. Reference: Park, S. J. et al. Astrocytes, but not microglia, rapidly sense H(2)O(2)via STAT6 phosphorylation, resulting in cyclooxygenase-2 expression and prostaglandin release. J Immunol 188, 5132-5141, doi:10.4049/jimmunol.1101600 (2012).

Kindlin3: Abcam provides several references for validation. We validated by western blot analysis of for K3 positive and K3 knockout cells. Ref: Canault, M. et al. Human CalDAG-GEFI gene (RASGRP2) mutation affects platelet function and causes severe bleeding. J Exp Med 211, 1349-1362, doi:10.1084/jem.20130477 (2014).

CD18: Abcam provides several references for validation. We validated by western blot analysis of with CD18 positive and CD18 knockout cells. Ref: Cullere, X., Lauterbach, M., Tsuboi, N. & Mayadas, T. N. Neutrophil-selective CD18 silencing using RNA interference in vivo. Blood 111, 3591-3598, doi:10.1182/blood-2007-12-127837 (2008).

GAPDH: Abcam provides several references for validation. We validated by western blot analysis. Ref: Valoskova, K. et al. A conserved major facilitator superfamily member orchestrates a subset of O-glycosylation to aid macrophage tissue invasion. Elife 8, doi:10.7554/eLife.41801 (2019).

B-Actin: Cell signaling provides several references for validation. We validated by western blot analysis.Ref: Rosenbaum, M. et al. Bcl10-controlled Malt1 paracaspase activity is key for the immune suppressive function of regulatory T cells. Nat Commun 10, 2352, doi:10.1038/s41467-019-10203-2 (2019).

α Tubulin: Santa Cruz provides several references for validation. Reference: Fichtman, B. et al. Combined loss of LAP1B and LAP1C results in an early onset multisystemic nuclear envelopathy. Nat Commun 10, 605, doi:10.1038/s41467-019-08493-7 (2019).

CD68: Abcam provides several references for validation. We validated by immunocytochemistry and immunohistochemistry i.e co-localization of staining with microglia in retina. Reference: 30862046Ye X et al. Oncogenic potential of truncated RXRa during colitis-associated colorectal tumorigenesis by promoting IL-6-STAT3 signaling. Nat Commun 10:1463 (2019).

Goat anti-Rabbit IgG (H+L) Cross-Adsorbed Secondary Antibody, Alexa Fluor 488: ThermoFisher Scientific provides several references for validation. Reference:Pittman, A. J., Law, M. Y. & Chien, C. B. Pathfinding in a large vertebrate axon tract: isotypic interactions guide retinotectal axons at multiple choice points. Development 135, 2865-2871, doi:10.1242/dev.025049 (2008).

Goat anti-Rabbit IgG (H+L) Highly Cross-Adsorbed Secondary Antibody, Alexa Fluor 647, ThermoFisher Scientificprovides several references for validation. Reference:Zalocusky, K. A. et al. Nucleus accumbens D2R cells signal prior outcomes and control risky decision-making. Nature 531, 642-646, doi:10.1038/nature17400 (2016).

Goat anti-Rabbit IgG (H+L) Cross-Adsorbed Secondary Antibody, Alexa Fluor 568: ThermoFisher Scientificprovides several references for validation. Reference:Lalit, P. A., Rodriguez, A. M., Downs, K. M. & Kamp, T. J. Generation of multipotent induced cardiac progenitor cells from mouse fibroblasts and potency testing in ex vivo mouse embryos. Nat Protoc 12, 1029-1054, doi:10.1038/nprot.2017.021 (2017).

Anti-rabbit IgG, HRP-linked Antibody, Cell Signaling Technology provides several references for validation. Reference:Wang, L. et al. ASCL1 is a MYCN- and LMO1-dependent member of the adrenergic neuroblastoma core regulatory circuitry. Nat Commun 10, 5622, doi:10.1038/s41467-019-13515-5 (2019).

Anti-mouse IgG, HRP-linked Antibody, Cell Signaling Technology provides several references for validation. Reference:Andersen, J. L. et al. Dimethyl fumarate is an allosteric covalent inhibitor of the p90 ribosomal S6 kinases. Nat Commun 9, 4344, doi:10.1038/s41467-018-06787-w (2018).

Eukaryotic cell lines

Policy information about cell lines

Cell line source(s)	RAW 264.7 was purchased from ATCC specifically for this project
Authentication	The RAW 264.7 cell line were commercially purchased from ATCC that guarantees authentication using Short Tandem Repeat (STR) Profiling, Cellular Morphology, Karyotyping and Cytochrome C Oxidase I (COI) Assay Testing
Mycoplasma contamination	The cell line was free of mycoplasma contamination as certified by ATCC. No further testing was done during the short period of study with these cells.
Commonly misidentified lines (See ICLAC register)	No commonly misidentified cell lines were used in the study.

Animals and other organisms

Policy information about studies involving animals; ARRIVE guidelines recommended for reporting animal research

Laboratory animals	WT: Mus musculus, C57BL/6, males and females, age postnatal day 6 to 60<br>Cx3cr1GFP/GFP: Mus musculus, C57BL/6, males and females, age postnatal day 6 to 60<br>Cx3cr1-cre (inducible):Mus musculus, C57BL/6, males and females, age postnatal day 6 to 60<br>TGF β1 flox/flox: Mus musculus, C57BL/6, males and females, age postnatal day 6 to 60<br>Integrin β1 flox/flox: Mus musculus, C57BL/6, males and females, age postnatal day 6 to 60<br>CD18 hypomorph: Mus musculus, C57BL/6, males and females, age postnatal day 6 to 60
Wild animals	No wild animals were involved in this study
Field-collected samples	No field-collected samples were used in the study
Ethics oversight	Animal experimental procedures were performed in accordance with National Institutes of Health (NIH) guidelines on animal care and all protocols were approved by the Institutional Animal Care and Use Committee at the Cleveland Clinic.

Note that full information on the approval of the study protocol must also be provided in the manuscript.
